# Can the Pulp Be Anesthetized by Injecting Cocain into the Peridental Tissues?

**Published:** 1902-10-15

**Authors:** T. W. Brophy


### CAN THE PULP BE ANESTHEITZED BY INJECTING COCAINE INTO THE PERIDENTAL TISSUES?

BY T. W. BROPHY, M.D., D.D.S.

It has been known for some time that though we remove a large part of the bone surrounding a tooth, except the pericementum, the pulp would remain alive. We frequently find that we can remove necrotic bone and leave the tooth covered only by the pericementum, and yet the pulp will remain alive. All our anatomists teach that the branches given off from the inferior dental artery and nerve are in direct communication with the pulp. This question has been under discussion for many years. Professor Cryer, of Philadelphia, has attempted to demonstrate that branches of the inferior dental nerve and inferior dental artery pass directly into the apical foramen to supply the tooth pulp. That has since been demonstrated as being untrue, and yet the books teach it in the old way. I believe Dr. Carpenter's statement is absolutely true, that if you can produce anesthesia of the pericementum by carrying an agent, capable of producing local anesthesia, down in around the neck of a tooth, between the gum and the tooth, down as far as you can, close to the alveolar border, bringing it into contact with the pericementum, you can anesthetize the tooth pulp.

Some gentlemen have raised the question as to whether the cocaine injected into the pericementum at the orifice of the alveoli would not go into the large arteries. No, that would not occur. You might get the agent in contact with minute arteries, and then into the capillaries and thus through the circulation and produce an anesthesia of the pulp by reason of the rich vascular supply of the pericementum and its direct communication with the minute vessels that enter the tooth foramen. When the new works on anatomy are written by men who know this subject, they will no longer make use of these very artistically prepared drawings, especially in reference to the tooth supply, but will make them from carefully prepared microscopic specimens. They will no longer show that every tooth pulp has its own artery and nerve supply.

The late Dr. Frank Abbott, of New York, whose memory we all honor, because of his tremendous energy and work for the advancement of the dental profession, made some microscopic specimens which demonstrated that the tooth pulp was not supplied in the manner as described in text-books, but through a plexus of vessels and nerves more nearly approaching in form the placenta. That plexus lies within the pericementum around the tooth root, and through that medium the pulp receives its nutrition. If the pericementum is destroyed the pulp dies, but the pulp may be destroyed and the pericementum will not die. Devitalize the pulp, and carry the preparation deep enough so as to involve the vessels surrounding the tooth root, and the pericementum will die to some extent. We see ends of roots around which there is no membrane because perhaps the arsenic has been carried up too far and passed out of the foramen, destroying the vitality of the tissue, and hence we have the end of the root extending up into a carious cavity, but the pericementum, being highly vascular, has been able to resist the destructive agent, and only a portion of it has been destroyed. We have sufficient adhesion of pericementum to cementum, and to the surrounding bony wall, to nourish the cementum and hold the tooth in place, to serve the purpose for which is was made. The statement made by Dr. Carpenter, that we may produce complete anesthesia of the pulp by applying agents around it which anesthetize the percimentum, is absolutely true.

I am not an advocate of the indiscriminate use of cocaine. If we use it at all, we must use it with extreme care. We really do not know what cocaine does in all cases. I have yet to read an article on cocaine that clearly convinces me that the writer knows just what it does. We know that it possesses some power to produce anesthesia, but we cannot say just how much is required in all cases to produce that effect, and yet not produce any toxic manifestations. Hence cocaine is a dangerous thing, even in the hands of the most skilled practitioner. It produces great depression of the heart, and the nerve centers of the brain if carried to the point of poisoning. Some gentlemen produce sufficient anesthesia with a one-half per cent solution to extract teeth, and seem to get along without any trouble. I have always held that whenever any infection occurs following the use of cocaine, it is not due to the cocaine. It is the instrument which was used, perhaps through the lack of thorough antiseptic cleanliness on the part of the operator. I very frequently have patients come to me suffering from necrosis of the jaws, either partial or complete, in which extensive areas of bone have been destroyed, because of the carelessness and lack of cleanliness of instruments on the part of the operator. These are the things we have to contend with all the time, and what dentists need to do to-day is to get down to the facts about cocaine.

Dr. Carpenter has read an excellent paper on cocaine and its use. Some gentlemen do not believe in cocaine at all, but there is no reason why we should not all know something about this matter. The thing to do is to make a thoroughly scientific investigation of this agent by a committee of men eminently fitted for this work, so that we can tell just what we can do with cocaine, and do it successfully, and without any danger, and to have the danger attending its use so clearly pointed out that we can not make a mistake.

I believe I have more evidences of the evils arising from the use of peroxide of hydrogen and similar agents than I have from the use of cocaine. This agent can be carried down into the tooth and into the root canals and set up a condition of things that is perfectly appalling. Some men put it into fistulous openings through the alveolar processes and gums, the peroxide comes into contact with pus, which often lies between the pericementum and bone and carries the infection along the border of the bone, setting up a complication worse than the original trouble. They put it into an antrum containing pus, or into a sinus. By reason of the rapid evolution of gas, an immense amount of harm is done, and the infection is forced into regions where it could not get if this agent had not been employed. Somebody will read a paper on some new agent, we think it is all right and we use it, using it in places where it should not be used at all.

Peroxide is all right in its proper place, but it is so often used in places where it does much harm. For this reason I have trespassed on your time to sound a note of warning that if you can stop the careless and injudicious use of these agents you will be doing your patients more good than by anything you can do in your office in the matter of caring for their teeth.—*Review*.

